# Interference Effects of Commercial Persistent Luminescence Materials on Rice Germination and Seedling Growth

**DOI:** 10.3390/plants12132554

**Published:** 2023-07-05

**Authors:** Nina Zhu, Xinpei Wei, Jingbo Yu, Shuo Zhang, Die Hu, Ping Li, Yunfei Xia, Kai Song

**Affiliations:** School of Life Science, Changchun Normal University, Changchun 130032, China; zhunina@ccsfu.edu.cn (N.Z.); weixinpei726@163.com (X.W.); yu8trista@126.com (J.Y.); zhangshuo727720@163.com (S.Z.); hudie18784775519@163.com (D.H.); chunlps@163.com (P.L.); xiayunfei1103@163.com (Y.X.)

**Keywords:** persistent luminescence materials, rice, germination, seedling growth, oxidative stress response

## Abstract

Persistent luminescence materials (PLMs) are widely used across a multitude of fields due to their distinct optical properties. However, like other micron-sized materials such as microplastics, the production and recycling processes of PLMs can lead to their accumulation in soil and water, potentially posing detrimental effects on plant growth and development. In this study, we investigated the impact of commercially available blue PLM (bPLM), green PLM (gPLM), and red PLM (rPLM) on germination, seedling growth, and oxidative stress responses in rice. Our findings demonstrate that the morphology and size of PLMs do not significantly differ in their effects on rice growth. All three types of PLMs significantly inhibited root length and stem length, disrupted root cell structures, and decreased seedling biomass. Interestingly, gPLM and bPLM were found to stimulate the synthesis of osmolytes and chlorophyll in rice, while rPLM had the opposite effect. Changes in the antioxidant enzyme system in rice clearly indicated that the three types of PLMs induced reactive oxygen species (ROS) damage in rice. This study enhances our understanding of the potential environmental impacts of PLMs, offering valuable insights for the safe and responsible use of these materials in various applications.

## 1. Introduction

Much like widely studied ceramics and microplastics, persistent luminescence materials (PLMs) represent a commonplace category of micrometric synthetic functional materials within our everyday environment. These primarily encompass compounds such as zinc sulfide, calcium sulfide phosphor, and strontium aluminate. The generation of various phosphorescence colors can be achieved by doping these materials with metallic ions, including copper, silver, and manganese. Their inherent chemical stability permits them to maintain their luminescent properties under diverse temperature and humidity conditions. In terms of physical properties, electrons within these materials transition and accumulate in “traps” upon excitation, subsequently being released gradually over time, thereby enabling a persistent luminescence. Their applications span a wide range of fields, including traffic signage, entertainment, lighting, textiles, coatings, agriculture, and biomedicine [1,2,3,4,5]. However, many commercialized PLMs, such as SrAl_2_O_4_:Eu^2+^, Dy^3+^ PLMs, exhibit a high sensitivity to water [6,7]. They undergo rapid hydrolysis reactions in water or humid environments, resulting in the formation of suspensions and precipitates [8]. This characteristic renders PLMs more susceptible to release into the environment during their handling, usage, and recycling processes, leading to their accumulation in soil or water bodies.

Micro- and nanoparticles, due to their extensive utilization, are susceptible to weathering, wear, and aging processes. These processes can lead to their gradual exposure and subsequent release into the environment, thereby posing a potential threat to terrestrial ecosystems. Primarily, the release and accumulation of these micro- and nanomaterials can instigate alterations in the properties and structure of the soil. This, in turn, can have significant implications for the growth of plant roots and the activity of rhizosphere microorganisms [9,10]. Furthermore, the transmission and translocation of micro- and nanomaterials within soil and water bodies may induce shifts in species competition and ecological niches among plants. Lastly, the absorption and accumulation of micro- and nanomaterials could potentially influence the interactions between plants and other organisms, mediated by their transmission along the food chain [9]. This sequence of events underscores the potential ecological implications of nanomaterial usage and the importance of monitoring their environmental impact [11].

Li et al. [12] conducted a study on the distribution and migration of microplastics (MPs) in edible plants. Their results demonstrate that polystyrene MPs of 0.2 μm are captured by an extracellular polysaccharide in lettuce cells, which then enter the plant and traverse the intercellular spaces via an apoplastic route. Subsequently, these MPs are translocated from the roots to the stems and leaves through the vascular system, driven by transpiration. In addition, it has been found that 4.8 μm MPs can physically obstruct the seed coat after 8 h of exposure, significantly inhibiting the germination of celery seeds. The germination rate was found to be 17% lower than the control [13]. Jiang et al. [14] discovered that MPs of 5 μm in different concentrations can inhibit the growth of broad bean roots, reduce biomass, and decrease catalase activity. Polyethylene microbeads did not affect the growth rate and photosynthetic pigment content of duckweed leaves but significantly impacted root growth through mechanical obstruction [15]. They can also adsorb onto the surface of duckweed, thereby entering the food chain [16]. However, contrary research findings also exist. For instance, Luo et al. [17] found that MPs at a concentration of 1.6 g/L significantly inhibited the photosynthetic activity of Chlorella cells.

While research on PLMs has primarily focused on their fabrication and applications [18,19,20], studies investigating the impact of PLMs on plants are notably scarce. Therefore, this study aims to fill this knowledge gap. We selected the most commonly used red (rPLM), green (gPLM), and blue (bPLM) photoluminescent materials in the environment as our research subjects, exploring their effects on the morphogenesis and physiological and biochemical indicators of rice. By gaining a deeper understanding of the interaction mechanisms between micromaterials and plants, we will be able to assess the potential application value of micromaterials in agricultural production, as well as their importance in improving plant stress resistance and conducting safety assessments.

## 2. Results and Discussion

### 2.1. Analysis of Physical Phase States

**Figure 1:**
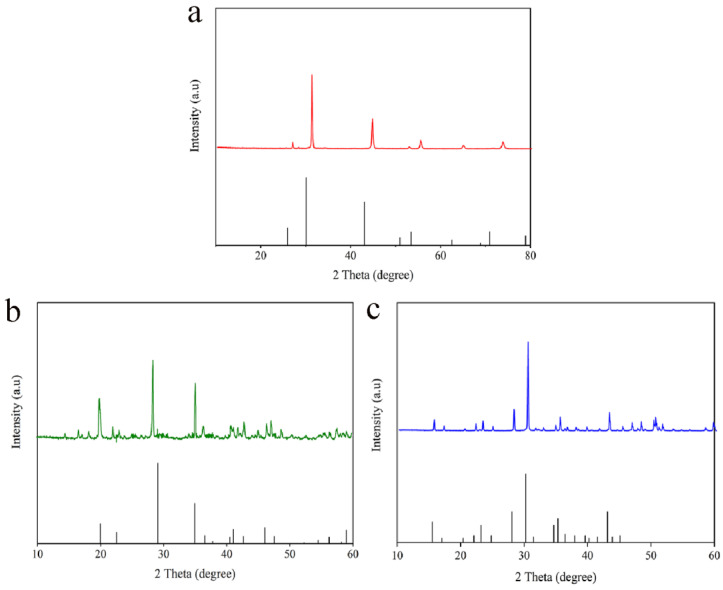
XRD patterns of rPLM (**a**), gPLM (**b**), and bPLM (**c**). Black lines represent the standard card atlas (rPLM, 01-075-0265 (Sr_.75_Ca_.25_S); gPLM, 00-031-1336(SrAl_2_O_4_); bPLM, 01-075-0265(Sr_.75_Ca_.25_S)).

The physical phase composition and lattice structure of PLMs can vary due to differences in their preparation processes. Notably, these structural variations often lead to changes in the physicochemical properties of the materials, which in turn can affect their interactions with plants [21,22,23,24,25]. As observed in Figure 1, the diffraction peak intensities of rPLM and bPLM align closely with the corresponding peak positions in the PDF standard cards 01-075-0265 (Sr_.75_Ca_.25_S) and 00-015-0016 (Sr_2_MgSi_2_O_7_), respectively. However, a slight leftward shift in the peak positions suggests that the interplanar spacing (D) of the material may have increased following the doping of Eu^2+^. In the case of gPLM, its diffraction peak positions and intensities largely coincide with those of the PDF standard card 00-031-1336 (SrAl_2_O_4_). Nevertheless, the appearance of some additional peaks indicates that the residues of Eu may have exerted some influence on the crystal structure, thereby causing minor alterations in the crystal structure.

**Figure 2:**
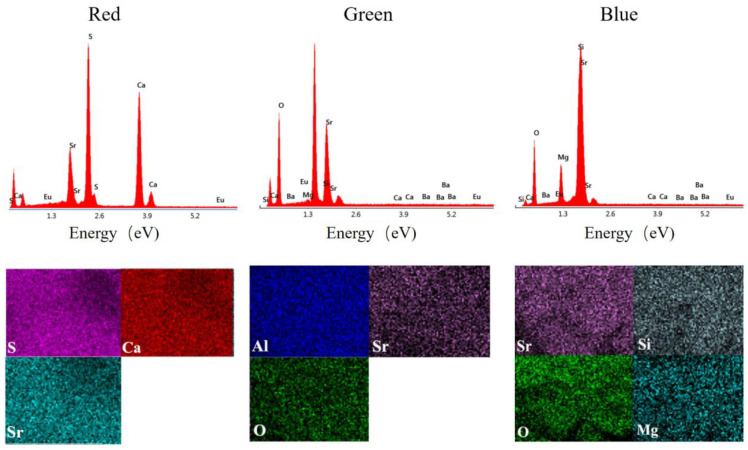
EDS spectrum under TEM of rPLM, gPLM and bPLM. The elemental mapping diagrams of rPLM (S, Ca, Sr), gPLM(Al, Sr, O), and bPLM (Sr, Si, O, Mg) are shown below, indicating a uniform distribution of the elements.

An energy-dispersive X-ray spectroscopy (EDS) analysis was also performed on the three types of PLMs, as shown in Figure 2. The analysis revealed that the primary elements in rPLM are sulfur (S), calcium (Ca), and magnesium (Mg); in bPLM, they are aluminum (Al), strontium (Sr), and oxygen (O); and in gPLM, they are strontium (Sr), silicon (Si), oxygen (O), and magnesium (Mg). Notably, in addition to the matrix elements, a trace signal of europium (Eu) was detected in the energy spectrum, further confirming the doping of Eu^2+^ ions in the matrix, which act as luminescent centers. This finding is consistent with the aforementioned XRD results, providing compelling evidence for the crucial role of Eu^2+^ ions in the material.

### 2.2. Morphological and Granulometric Analysis

**Figure 3:**
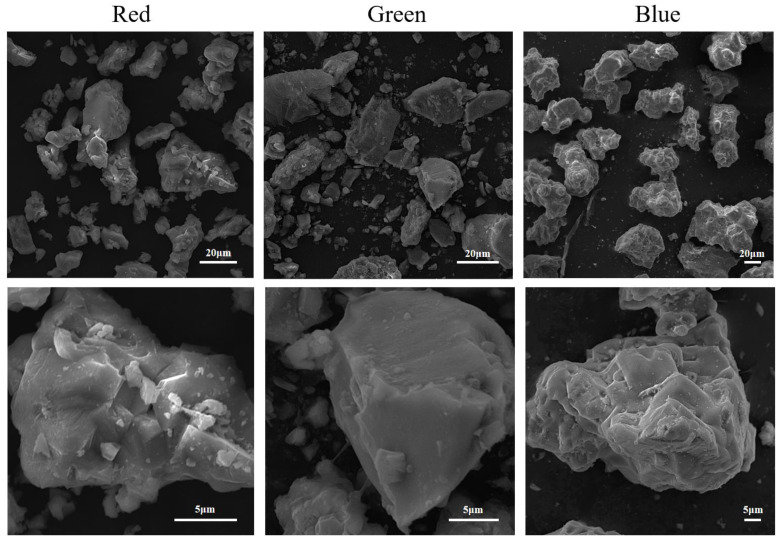
SEM images of rPLM, gPLM, and bPLM.

Existing research has established that the morphology, scale, and physicochemical properties of micro- and nanomaterials can all exert influence on plants [26,27,28,29,30,31]. As depicted in Figure 3, both rPLM and bPLM exhibit a relatively rough surface and an irregular blocklike structure, with a nonuniform particle size distribution. Specifically, the particle size of rPLM is predominantly around 30 μm, while that of bPLM is primarily around 40 μm. In contrast, gPLM has a comparatively smooth surface, resembling a sharply edged tetrahedron, with an average particle size of 15 ± 3 μm.

Furthermore, the surface charge characteristics of materials may also influence their interactions with plants to a certain extent. For instance, Sergey et al. found that positively charged iron oxide nanomaterials exhibited a more pronounced inhibitory effect on the growth of Arabidopsis thaliana seedlings (20% inhibition for positive charge, 3.6% for negative charge) and root length (48% inhibition for positive charge, negligible for negative charge) compared with negatively charged nanoiron oxide. Table 1 summarizes the data on the morphology, particle size, and zeta potential of the three types of PLMs.

### 2.3. Spectral Analysis

**Figure 4:**
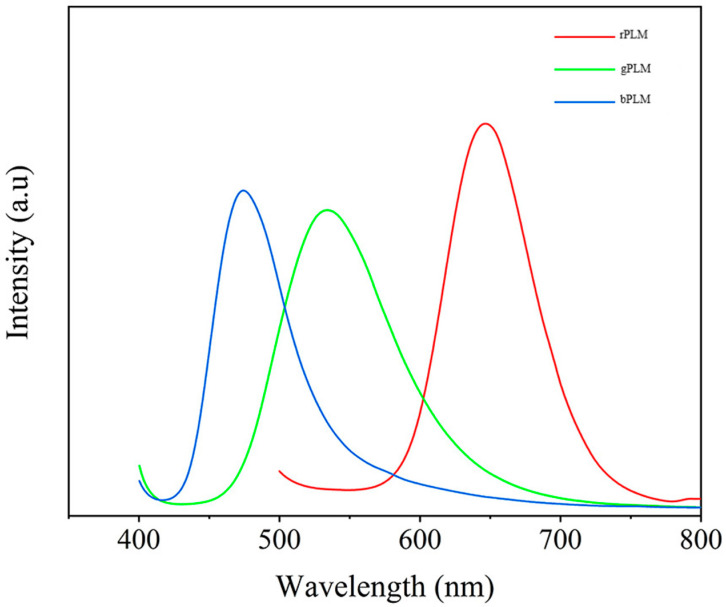
Emission spectra of rPLM, gPLM, and bPLM under 365 nm excitation light.

As depicted in Figure 4, rPLM exhibits a strong broadband emission in the 550 to 720 nm range, with the most intense peak located at 645 nm. gPLM shows a broadband emission in the 470 to 620 nm range, with its strongest emission peak at 534 nm. bPLM has an emission wavelength range between 420 and 560 nm, reaching its maximum emission peak at 474 nm. The emission ranges of these three types of PLMs are consistent with data reported in the relevant literature.

### 2.4. Effects on Rice Seed Germination

**Figure 5:**
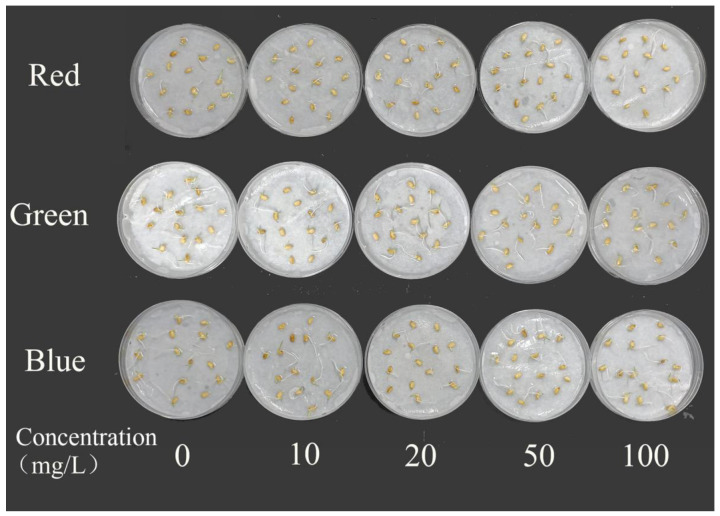
Photographs of rice seed germination under series concentrations of the three PLM treatments.

As shown in Table 2, there were no statistically significant differences (*p* > 0.05) in seed germination capacity and germination rate between the control group (0 mg/L) and seeds treated with different concentrations of PLMs solutions. Overall, there were also no statistically significant differences (*p* > 0.05) in the germination capacity and germination rate of rice seeds treated with the three different colored PLM solutions. Figure 5 illustrates the germination of rice seeds under treatment with the three types of PLM solutions.

### 2.5. Effects on Seedling Roots and Stems

**Figure 6:**
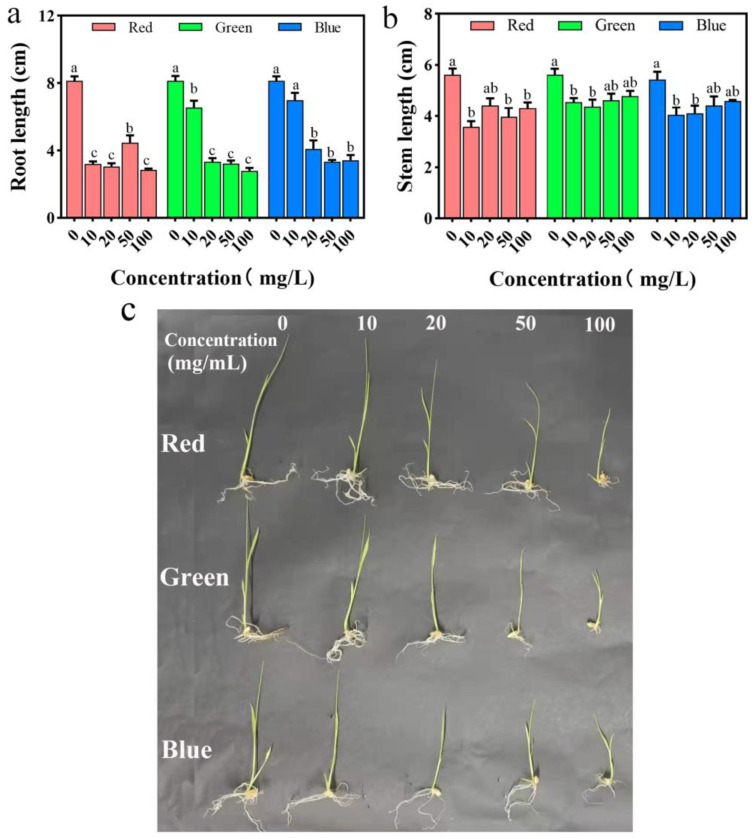
Effects of varying concentrations of rPLM, gPLM, and bPLM on the root length (**a**) and stem length (**b**) of rice seedlings. These values are expressed as the mean ± standard deviation (SD) of the samples. The comparison within groups was expressed by lowercase letters, and significant difference was defined with different letters (*p* < 0.05). Photographs of morphological changes in rice seedlings (**c**) exposed to different concentrations of rPLM, gPLM, and bPLM for 10 days. It is clearly shown above that the growth of rice was significantly inhibited with increasing concentrations of PLMs.

Roots and stems constitute the primary nutritional organs of plants, with roots primarily responsible for the absorption of water and inorganic salts, among other nutrients, and stems functioning to transport and store these nutrients. The development of a plant’s root–stem system directly influences its growth condition. Figure 6 illustrates the impact of treating rice with different concentrations of three PLM solutions on root and stem length. As shown in Figure 6a, with an increase in PLM treatment solution concentration, all PLMs exhibit a significant inhibitory effect on the length of rice roots (*p* < 0.05). Specifically, when the concentration of rPLM and gPLM materials reaches 100 mg/L, their inhibitory effect is most pronounced, reducing root length to only 35.2% and 34.3% of the control group, respectively. Meanwhile, bPLM shows the most significant inhibitory effect at a concentration of 50 mg/L, reducing root length to only 40.8% of the control group. As depicted in Figure 6b, with an increase in treatment solution concentration, all three PLMs exhibit a significant inhibitory effect on the length of rice stems (*p* < 0.05). The inhibitory effect is most pronounced when the concentration of rPLM and bPLM reaches 10 mg/L, reducing stem length to only 63.7% and 74.8% of the control group, respectively. In contrast, gPLM shows the greatest inhibitory effect on rice stem length at a concentration of 20 mg/L, reducing stem length to only 78% of the control group. These phenomena have also been observed in studies on the inhibitory effect of polystyrene nanoparticles on the initial root length of rice [32]. Typically, the root system is a key part of a plant that senses changes in the external environment and adapts its structure accordingly [33]. The significant inhibition of rice root length by PLM provides a direct reflection of PLM’s phytotoxicity to rice (Figure 6c).

### 2.6. Impacts on Root Cell Morphology

**Figure 7:**
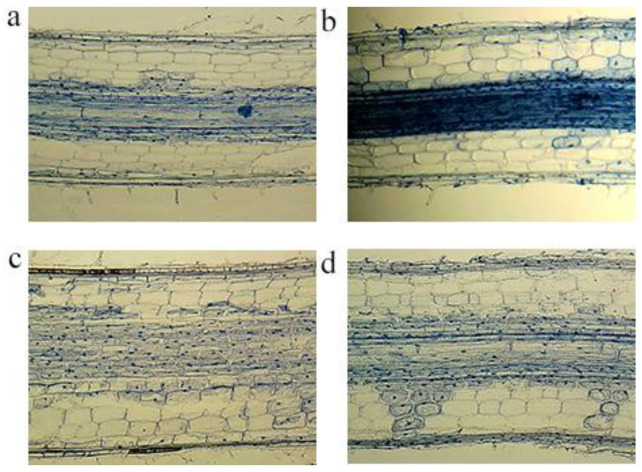
Optical micrographs of longitudinal sections of mature root zones of control (**a**) and rice seedlings treated with 100 mg/L rPLM (**b**), gPLM (**c**), and bPLM (**d**).

To elucidate the mechanisms underlying the impact of PLMs on rice root system, the mature zones of the rice seedlings’ root system treated with 100 mg/L of the three PLMs were selected for longitudinal section observation. As depicted in Figure 7, compared with the control group, the mature zones of the rice roots treated with 100 mg/L of PLMs showed sparse root hairs, a decrease in the cell layers of the cortex, and a morphological transition of cells from rectangular to irregularly elliptical. These observations suggest that the structure of the mature zone of the root is altered under PLM treatment, potentially disrupting the absorption of nutrients and leading to developmental delays in rice, aligning with the findings of previous studies [34].

### 2.7. Impact on Root Vitality and Cell Membrane System

**Figure 8:**
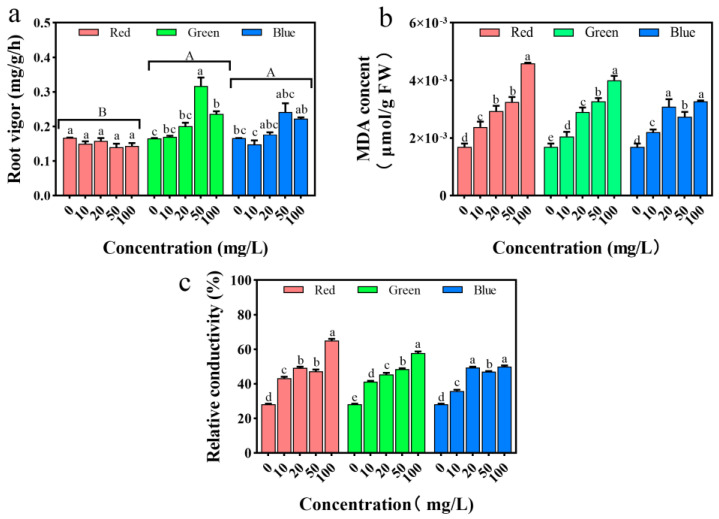
Changes in root activity of rice seedlings (**a**), cellular MDA content (**b**), and relative electrical conductivity of rice cells (**c**) treated with different concentrations of rPLM, gPLM, and bPLM. The values are given as mean ± SD of the samples. The comparison between groups is expressed by uppercase letters, while the comparison within groups was expressed by lowercase letters, and significant difference was defined with different letters (*p* < 0.05).

Root vitality refers to the capacity of a plant’s root system to absorb nutrients and to synthesize and convert organic matter. Greater root vitality is associated with stronger metabolic activity in the plant’s root system [32]. Under treatment with gPLM and bPLM, the root vitality of the seedlings increased significantly (*p* < 0.05), with the most pronounced promotion effect observed at a solution concentration of 50 mg/L. In particular, the root vitality of seedlings treated with gPLM was increased by 91.6% compared with the control group, while that of seedlings treated with bPLM was elevated by 34.0% (Figure 8a).

Malondialdehyde (MDA) is an important indicator for assessing the degree of lipid peroxidation in plant cells. When plants are subjected to external stress that generates an excess of reactive oxygen species (ROS), a large number of free radicals can act on lipids, causing oxidative damage. The final oxidation product obtained is MDA. Ibrahim et al. found that the MDA content in rice seedlings treated with high concentrations (above 30 mg/L) of CuO nanoparticles was higher. Here, with the increase in PLM solution concentration, the MDA content in the rice seedlings significantly increased (*p* < 0.05) (Figure 8b). Notably, the MDA content in rice seedlings treated with rPLM solution increased by an average of 94.52% compared with the control. Although there was no significant difference (*p* > 0.05) between the three PLM treatment groups according to the statistical analysis, all of them caused severe lipid peroxidation in rice cells.

When plants are subjected to external stress, damage to the cell membrane can cause electrolytes in the cell to exude, increasing the electrical conductivity of the cell. Therefore, to further investigate the impact of PLM on rice cells, we measured the electrical conductivity of rice leaves. As shown in Figure 8c, the relative electrical conductivity of rice leaves treated with PLM significantly increased (*p* < 0.05), and it reached its highest at a concentration of 100 mg/L, which was 1.05 times, 1.31 times, and 0.77 times that of the control group, respectively.

### 2.8. Impact on Seedling Biomass

**Figure 9:**
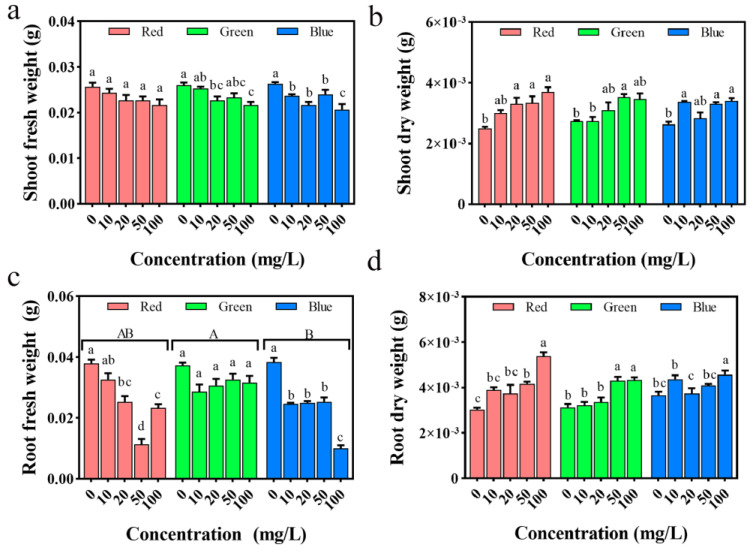
Changes in shoot fresh weight (**a**), shoot dry weight (**b**), and root fresh weight (**c**) and root dry weight (**d**) of rice seedlings treated with serial concentrations of rPLM, gPLM, and bPLM. These values are expressed as the mean ± SD of the samples. The comparison between groups is expressed by uppercase letters, while the comparison within groups was expressed by lowercase letters, and significant difference was defined with different letters (*p* < 0.05).

Plant biomass alterations serve as a fundamental indicator for assessing the degree of plant stress. As depicted in Figure 9a,c, with an escalation in PLM concentrations, both the above-ground and below-ground fresh weight of rice seedlings tends to decrease. Under rPLM treatment, while a declining trend is observed in the above-ground fresh weight of seedlings, the decrease does not reach a significant level (*p* > 0.05). gPLM treatment results in a reduction in the below-ground fresh weight of seedlings, but the inhibitory effect is likewise not significant.

Contrastingly, as illustrated in Figure 9b,d, the dry weight of both the above-ground and below-ground parts of rice seedlings shows an upward trend with the increasing PLM concentration. When rPLM concentration reaches 100 mg/L, the dry weight of the above-ground and below-ground parts of seedlings, respectively, increases by 32.4% and 43.8% compared with the control group. At 50 mg/L gPLM, the dry weight of the above-ground and below-ground parts of seedlings rises by 29.3% and 37.2% compared with the control. With bPLM solution at a concentration of 100 mg/L, the dry weight of the above-ground and below-ground parts of seedlings, respectively, rises by 29.1% and 24.5% compared with the control. This parallels research showing that CuO nanoparticles reduce the fresh weight of rice roots and leaves, exhibiting a dose-dependent toxic effect [35].

**Figure 10:**
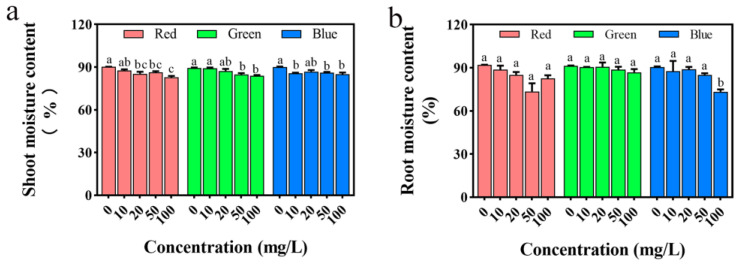
Effects of treatments with varying concentrations of rPLM, gPLM, and bPLM on shoot (**a**) and root moisture content (**b**) of rice seedlings. The values are given as mean ± SD of samples. The comparison within groups was expressed by lowercase letters, and significant difference was defined with different letters (*p* < 0.05).

According to the moisture content of the above-ground and below-ground parts of rice seedlings (Figure 10), compared with the control group, the moisture content of the above-ground parts of seedlings in all exposure groups gradually declines. Specifically, the moisture content in the above-ground parts of seedlings under the treatment of PLM solutions at 10, 20, 50, and 100 mg/L decreases by 2.93%, 5.47%, 4.40%, and 8.20%, respectively, compared with the control group. In the case of gPLM treatment at 10, 20, 50, and 100 mg/L, the moisture content in the above-ground parts of seedlings drops by 0.30%, 2.46%, 5.24%, and 6.10%, respectively, compared with the control. For bPLM treatment at 10, 20, 50, and 100 mg/L, the moisture content in the above-ground parts of seedlings falls by 4.69%, 3.46%, 4.19%, and 5.50%, respectively, compared with the control. This suggests that all three PLMs inhibit the moisture content in the above-ground parts of rice seedlings in a dose-dependent manner.

### 2.9. Impact on Seedling Antioxidant System

Under normal conditions, reactive oxygen species (ROS) within plants maintain a balance. However, when plants are subjected to external stimuli, accelerating the production of ROS, the plant itself will initiate its antioxidant system to remove excess ROS. Superoxide dismutase (SOD), catalase (CAT), and peroxidase (POD) are the three main antioxidant enzymes in this system. SOD catalyzes the conversion of superoxide anions in plants into H_2_O_2_ and O_2_ [36].

**Figure 11:**
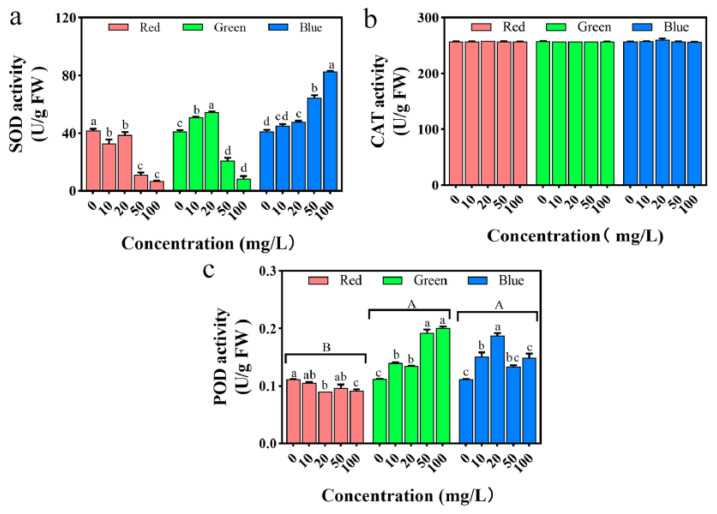
Effect of treatments with different concentration of rPLM, gPLM, and bPLM on the activities of SOD (**a**), CAT (**b**), and POD (**c**) in rice seedlings. The values are given as mean ± SD of samples. The comparison between groups is expressed by uppercase letters, while the comparison within groups was expressed by lowercase letters, and significant difference was defined with different letters (*p* < 0.05).

As shown in Figure 11a, with the increase in treatment liquid concentration, the activity of the SOD enzyme in rice seedlings treated with PLM significantly decreases (*p* < 0.05). The SOD enzyme activity in rice treated with gPLM shows an initial increase followed by a decrease, while the activity of the SOD enzyme in rice treated with bPLM significantly increases (*p* < 0.05). This suggests that all three PLMs lead to the production of a large amount of ROS in rice. However, the ROS in rice induced by a low concentration gPLM solution and gPLM treatment promotes the expression of a large amount of the SOD enzyme, thereby removing excess ROS. The increase in ROS in rice treated with rPLM and a high concentration of gPLM far exceeds the clearance capability of SOD, leading to metabolic imbalance and a decrease in SOD enzyme activity.

CAT and POD represent the second line of defense in ROS removal. They can catalyze the conversion of H_2_O_2_ into H_2_O and O_2_, and once H_2_O_2_ is overproduced due to abiotic stress or the SOD defense system, CAT and POD will be activated to remove H_2_O_2_. As shown in Figure 11b, the activity of the CAT enzyme in seedlings treated with the three PLMs shows no significant difference compared with the control group (*p* > 0.05). As shown in Figure 11c, the activity of POD in rice treated with rPLM gradually decreases. This corresponds to the earlier changes in SOD activity, further proving that rPLM has a stronger toxic effect on rice. However, the activity of POD in rice treated with gPLM shows an increasing trend. Under different concentrations of gPLM treatment, the vitality of rice POD first increases and then decreases, which may be related to the fact that SOD fails to completely remove excess ROS in rice.

### 2.10. Impact of H_2_O_2_ Accumulation

Hydrogen peroxide (H_2_O_2_) serves as a key representative of ROS. At moderate levels, H_2_O_2_ functions as an intracellular signaling molecule, contributing to plant defense against bacterial invasions and bolstering plant immunity. However, high concentrations of H_2_O_2_ can inflict oxidative damage on major biological macromolecules, leading to protein and DNA impairment.

**Figure 12:**
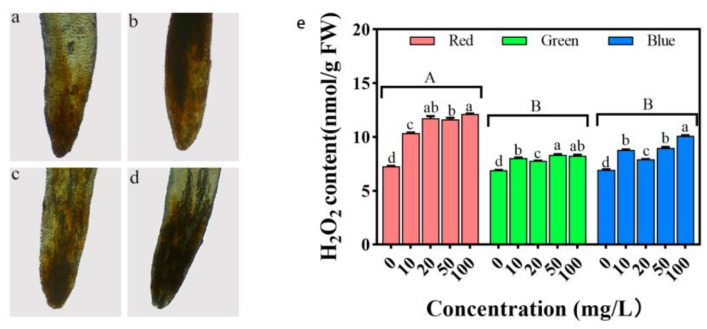
DAB-stained images of rice leaves without treatment (**a**) and under treatment with 100 mg/L rPLM (**b**), gPLM (**c**), and bPLM (**d**) solutions. Changes in H_2_O_2_ content in rice leaves treated with different concentrations of rPLM, gPLM, and bPLM. These values are the mean ± SD of the samples (**e**). The comparison between groups is expressed by uppercase letters, while the comparison within groups was expressed by lowercase letters, and significant difference was defined with different letters (*p* < 0.05).

Given this dual role of H_2_O_2_, we aimed to understand the oxidative stress imposed by three variants of PLM on rice seedlings. We selected rice leaves subjected to treatment with the prototypic concentration of 100 mg/L PLM. We applied DAB staining to these samples to evaluate H_2_O_2_ production (Figure 12a–d).

Our analysis reveals a wide range in H_2_O_2_ content across treatments. When the PLM concentration was held at 100 mg/L, rPLM treatment exhibited the peak hydrogen peroxide level at 12.21 nmol/g. In contrast, gPLM treatment displayed the lowest hydrogen peroxide concentration, amounting to a mere 8.27 nmol/g (Figure 12e).

### 2.11. Impact on Seedling Osmotic Regulatory Substances

**Figure 13:**
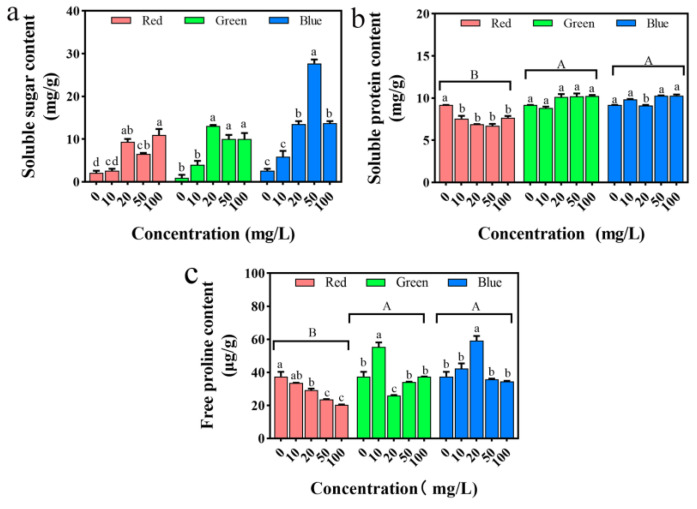
Changes in soluble sugars (**a**) and soluble protein (**b**) and free proline content (**c**) in rice seedlings treated with different concentrations of rPLM, gPLM, and bPLM. These values are the mean ± SD of the samples. The comparison between groups is expressed by uppercase letters, while the comparison within groups was expressed by lowercase letters, and significant difference was defined with different letters (*p* < 0.05).

Soluble sugar can provide the energy required for various life activities of plants and can also help plants resist external stress. In addition, soluble protein can maintain the metabolism of plants and improve their resistance. As can be seen from Figure 13a, compared with the control treatment, the soluble sugar content in the treatment groups of the three PLMs significantly increased (*p* < 0.05). When the concentration of the treatment solution is 20 mg/L, the increase in soluble sugar content is most noticeable under gPLM treatment, increasing more than 10 times compared with the control group; when the concentration of the treatment solution is 50 mg/L, the increase in soluble sugar content is most noticeable under bPLM treatment, increasing more than nine times compared with the control group; when the concentration of the treatment solution is 100 mg/L, the increase in soluble sugar content is most noticeable under rPLM treatment, increasing more than four times compared with the control group. The three PLMs induced the plant’s stress resistance behavior, continuously increasing the soluble sugar content to improve the stress resistance of the rice itself. As can be seen from Figure 13b, compared with the control group, rPLM significantly inhibits the accumulation of soluble protein in seedlings (*p* < 0.05). When the concentration of the solution is 50 mg/L, the soluble protein content is the smallest, only 6.71 ± 0.35 mg/g. However, gPLM and bPLM have promoted the accumulation of soluble protein in the seedlings. But, the promoting effect of both on the soluble protein content in rice has no significant difference (*p* > 0.05).

As shown in Figure 13c, the free proline in rice leaves under rPLM treatment significantly decreases at concentrations of 20~100 mg/L (*p* < 0.05). Under gPLM and bPLM treatment, the free proline content first increases and then decreases, reaching peaks at 10 and 20 mg/L, respectively, which are 1.48 times and 1.58 times that of the control. This result is consistent with the research of Wang et al. This suggests that rice can alleviate the toxicity of gPLM and bPLM by regulating the osmotic potential in the body, enhancing its own resistance. However, with the increase in stress concentration, the synthesis of free proline is restricted, the osmotic regulation mechanism is destroyed, and the rice seedlings are damaged. It is generally believed that free proline under stress can interact with enzymes to maintain enzyme activity [35]. The change in the content of free proline in rice under PLM treatment also corroborates the changes in the antioxidant enzyme system mentioned earlier.

### 2.12. Impact on the Mineral Elements in Seedlings

**Figure 14:**
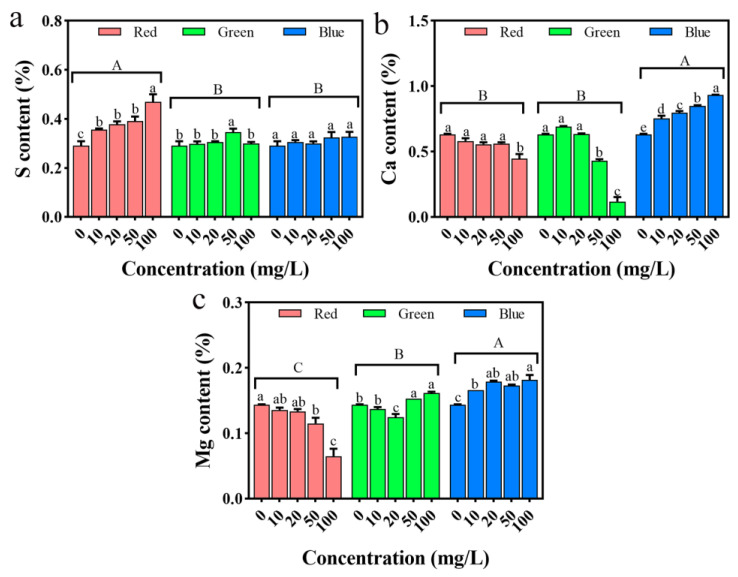
Changes in S (**a**), Ca (**b**), and Mg (**c**) content in rice seedlings treated with different concentrations of rPLM, gPLM, and bPLM. The values are given as mean ± SD of samples. The comparison between groups is expressed by uppercase letters, while the comparison within groups was expressed by lowercase letters, and significant difference was defined with different letters (*p* < 0.05).

By measuring the morphology and physiological and biochemical indicators of rice seedlings, it can be clearly shown that the three types of PLM have certain effects on rice, and it is speculated that this is related to the composition of PLM itself and the ions released by dissolution. Therefore, the content of three mineral elements, S, Ca, and Mg, in rice seedlings was analyzed. As shown in Figure 14, under rPLM stress, the content of S element in rice significantly increased (*p* < 0.05), reaching its highest at 100 mg/L, an increase of 65.14% compared with the control; the content of Ca and Mg showed a decreasing trend, reaching their lowest at 100 mg/L, a decrease of 29.42% and 23.68%, respectively, compared with the control. Under gPLM stress, the content of S element in rice only significantly increased at 50 mg/L (*p* < 0.05); the content of Ca showed a trend of first increasing then decreasing, with the highest at 10 mg/L, and significantly decreased at 50, 100 mg/L (*p* < 0.05); the content of Mg first decreased then increased, with the highest at 100 mg/L and the lowest at 20 mg/L. Under different concentrations of bPLM stress, the content of S did not change significantly (*p* > 0.05); the content of Ca and Mg gradually increased, reaching their highest at 100 mg/L, an increase of 47.82% and 26.56%, respectively, compared with the control.

### 2.13. Impact on Seedling Chlorophyll

**Figure 15:**
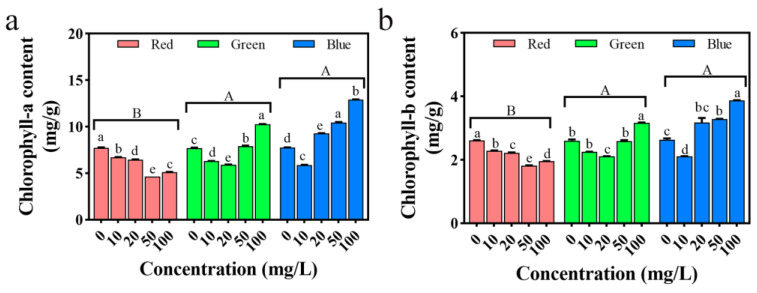
Changes in chlorophyll a (**a**) and chlorophyll b (**b**) contents in rice seedlings treated with different concentrations of rPLM, gPLM, and bPLM. The values are given as mean ± SD of samples. The comparison between groups is expressed by uppercase letters, while the comparison within groups was expressed by lowercase letters, and significant difference was defined with different letters (*p* < 0.05).

Photosynthetic pigments are considered important indicators for evaluating particle toxicity [37]. As shown in Figure 15, under rPLM treatment, the content of chlorophyll a and chlorophyll b in rice showed a significant downward trend (*p* < 0.05). Compared with the control group, the average content of chlorophyll a and chlorophyll b decreased by 25.69% and 20.70%, respectively. Under gPLM and bPLM treatment, the content of chlorophyll a and chlorophyll b in rice showed a trend of first decreasing and then increasing. At 20 mg/L of gPLM, the content of chlorophyll a and chlorophyll b was the lowest, only 5.91 ± 0.03 mg/g and 2.25 ± 0.01 mg/g, respectively. At 10 mg/L of bPLM, the content of chlorophyll a and chlorophyll b was the lowest, 5.88 ± 0.04 mg/g and 2.11 ± 0.01 mg/g, respectively.

## 3. Discussion

In this study, the impact of three types of PLMs on the germination of rice seeds was not significant, which contradicts previous reports. For instance, Dong et al. [38] found a decrease in the germination rate of rapeseed seeds treated with poly(methyl methacrylate) particles smaller than 10 μm. Guo et al. [39] also reported that polystyrene particles of 2 μm and 80 nm significantly inhibited the germination of herbaceous plant seeds. We speculate that this discrepancy may be due to the thick and robust seed coat of rice seeds, which can protect the seeds from adverse external conditions. Additionally, the selective absorption capacity of the seed coat might prevent particles with sizes ranging from 15–40 μm from penetrating the seed coat and entering the seed, thereby reducing the toxic effects of PLMs on rice seeds.

Certain studies have demonstrated that the growth-promoting effect of water-dispersible carbon nanomaterials on mung beans is closely related to their morphology. For instance, disk-shaped single-walled carbon nanotubes with diameters of less than 10 nm have been found to promote mung bean growth more effectively than straight, open-ended multiwalled carbon nanotubes with diameters of 180 nm. Meanwhile, carbon nanowhiskers with sizes between 150–200 nm have been shown to exert a lesser stimulatory effect on mung bean growth [40]. Although the morphology of gPLM is more uniform than that of rPLM and bPLM (see Figure 3), these morphological differences do not seem to have a significant impact on the inhibitory effect on rice root and stem length. This could be due to PLM particles exceeding the micrometer level causing root blockage, reducing nutrient absorption, and thereby inhibiting the growth of rice roots and stems (Figure 6c). This result is consistent with the changes in the morphology of rice root cells.

Despite the decrease in seedling root length under the stress of bPLMs and gPLMs, their influence stimulated an increase in root vitality to meet the nutrient absorption requirements of rice seedling growth. However, no significant difference (*p* > 0.05) was observed between the promotive effects of these two materials on seedling root vitality. In contrast, the root vitality of seedlings treated with rPLM did not increase but decreased. When the PLM concentration was 50 mg/L, root vitality was at its lowest, showing a reduction of 16.2% compared with the control. This could potentially be due to the presence of toxic sulfides in the matrix, which may have reduced nutrient absorption by the plant [41]. Furthermore, rPLM has a stronger toxic effect on rice seedlings, causing metabolic imbalance in rice, whereas the stress of gPLM and bPLM on rice has stimulated the accumulation of soluble protein to participate in plant osmotic regulation, thus maintaining their normal physiological activities [34]. The main matrix of rPLM is sulfides, and the mapping shows that S elements account for 43% of the composition (Figure 2). Research on the natural sulfur isotope abundance ratio of Scirpus maritimus shows that most of the sulfate in marsh plants comes from sulfides [42]. Even if the concentration of dissolved sulfides is very low, 50–96% of the S in plants still comes from different sedimentary sulfides [43]. The significant increase in S element in rice under rPLM stress further confirms the toxic sulfide effect on rice, leading to inhibited rice growth. The decrease in the content of Ca and Mg elements in rice is also due to sulfides. This is because the concentration of Ca and Mg in acidic sulfate soils is usually unsaturated, and the acidic substances produced by sulfide oxidation can cause the loss of Ca and Mg at the cation exchange sites in the rhizosphere [44].

All three PLMs similarly inhibit the fresh weight of both the above-ground and below-ground parts of rice seedlings, with the overall inhibitory effect intensifying with increased PLM concentrations. Notably, compared with the other two PLMs, only the inhibitory effect of bPLM treatment on the fresh weight of the below-ground parts of seedlings has statistical significance (*p* < 0.05). This may be due to the larger standard error in the experimental data of the below-ground fresh weight of seedlings treated with rPLM and gPLM. There is no significant impact on the moisture content in the below-ground parts of seedlings across the three PLMs (*p* > 0.05), except for a significant decrease of 17.90% compared with the control group in the case of bPLM treatment at 100 mg/L. Moisture content greatly influences plant growth. The overall moisture content of seedlings treated with different concentrations of PLM in this experiment declines, which corresponds with the earlier changes observed in rice roots and stems, further confirming that all three PLMs inhibit the growth of rice seedlings.

At the cellular level, the changes in relative electrical conductivity and MDA levels of rice cells confirm that all three types of PLMs can inflict damage on rice cells, leading to cell membrane impairment. Compared with plants treated with the control group, H_2_O_2_ accumulates more at the leaf tip after treatment with the three PLMs. At a concentration of 100 mg/L, the amount of H_2_O_2_ accumulated in the leaves is, in descending order, rPLM, bPLM, and gPLM, which is consistent with the trend of changes in POD activity. POD can reduce H_2_O_2_ to H_2_O, thereby removing excess H_2_O_2_ in plants. The POD activity in rice treated with rPLM is significantly lower than the control group (*p* < 0.05), unable to clear excess H_2_O_2_, thus resulting in significant accumulation of H_2_O_2_ in the rice leaves. However, the POD activity in rice treated with gPLM is the strongest; therefore, the amount of H_2_O_2_ accumulated in the leaves under this treatment is the least. Moreover, Ca ions can maintain the stability of the cell wall and cell membrane, and at the same time, regulate the antioxidant system of the plant, since SOD is a calmodulin-dependent enzyme. Therefore, the trend of changes in the content of Ca in rice seedlings under the stress of the three PLMs is similar to the trend of changes in SOD enzyme activity (Figure 11a), which is a response of rice seedlings to PLM.

Mg is a component of plant chlorophyll, and here, we speculate that the dissolution of Mg ions in gPLM and bPLM solutions may promote chlorophyll synthesis. Studies have shown that stress can reduce the content of photosynthetic pigments in rice, and the changing patterns of chlorophyll a and chlorophyll b are similar [32]. However, in this study, high concentrations of gPLM and bPLM had a promoting effect on the content of photosynthetic pigments in rice, which may be due to the dissolution of Mg ions in the aqueous solution. It is well known that Mg is an essential element for the formation of chlorophyll. It can promote the combination of the porphyrin ring and surrounding organic chains to form chlorophyll. After the Mg ions released by the dissolution of gPLM and bPLM are absorbed by the rice seedlings, the synthesis of chlorophyll in the body is accelerated, thereby increasing the content of chlorophyll a and chlorophyll b. This is also confirmed by the changes in the mineral elements in plants mentioned earlier.

## 4. Materials and Methods

### 4.1. Materials and Instruments

The rPLM, gPLM, and bPLM photoluminescent materials were procured from Dalian Luminescent Technology Co., Ltd., Dalian, China. The rice variety used was YangJing 687. Acetone, indantrione, glacial acetic acid, tris(phenyl)tetrazolium chloride, and o-phenylenediamine were purchased from Beijing Fine Chemicals Co., Ltd., Beijing China. Trichloroacetic acid, thiobarbituric acid, anthrone, sulfosalicylic acid, and Coomassie Brilliant Blue G-250 was obtained from Sinopharm Chemical Reagent Co., Ltd., Shanghai, China. Guaiacol was sourced from Shanghai Source Leaf Biotechnology Co., Ltd., Shanghai, China. Anhydrous ethanol and barium chloride were supplied by Xilong Scientific Co., Ltd., Shantou, China. All reagents were of analytical grade.

### 4.2. Experimental Methods

Characterization of the PLMs: The XRD of the PLMs was performed using an X-ray diffractometer (D2PHASER, Bruker, Singapore). The morphology of the PLMs was analyzed by a scanning electron microscope (VEGA3, TESCAN, Brno, Czech Republic). All photoluminescence spectra were measured on a Hitachi F-4500 fluorescence spectrophotometer equipped with a continuous 150 W Xe-arc lamp and a 10 mm quartz cuvette.

Processing and cultivation of rice seeds: Uniformly shaped and full-grained rice seeds were selected for this study. The seeds were first sterilized by soaking in a 75% ethanol solution. The sterilized seeds were then evenly placed at the bottom of a disposable petri dish on sterilized filter paper using sterile tweezers. Subsequently, aliquots of 5 mL of PLM suspensions with concentrations of 0 mg/mL, 10 mg/mL, 20 mg/mL, 50 mg/mL, and 100 mg/mL were, respectively, measured and slowly poured into petri dishes. Finally, the petri dishes were placed in a constant temperature incubator for cultivation, with the filter paper and PLM treatment solution replaced daily.

Measurement of germination indicators. Rice seeds began to germinate (white seed exposure) on the third day of cultivation and stopped germinating on the sixth day. We recorded the daily number of germinated seeds during this period to calculate the germination potential and germination rate of the seeds. We assigned 15 seeds to each treatment group and replicated this process three times.

Measurement of physiological indicators: After the complete germination of the rice seeds, we conducted another 7-day cultivation and seedling collection. A sanitized ruler was employed to measure the length of the primary crown root of the rice seedlings as well as the length from the base of the first full leaf to its tip. The treatment solution on the surface of the rice seedlings was wiped off using filter paper, followed by measuring the fresh weight of both the aboveground and underground parts. Subsequently, the weighed rice roots and stems were placed in a drying oven for desiccation, and the dry weights of the aboveground and underground parts were determined. Finally, the moisture content of the aboveground and underground parts was calculated. We assigned 30 seedlings to each treatment group and replicated this process three times.

Observation of root cell morphology: We used the paraffin sectioning method to process and section the mature root tissue of the rice seedlings and selected relatively complete tissue sections for observation under a biological microscope.

Measurement of basic biochemical indicators: In this study, we followed the method described in reference and used the tris(phenyl)tetrazolium chloride method to measure root vitality; the thiobarbituric acid method to measure MDA content; the nitroblue tetrazolium method to measure superoxide dismutase (SOD) activity; the guaiacol method to measure peroxidase (POD) activity; and the hydrogen peroxide method to measure catalase (CAT) activity. In addition, we used the anthrone method to measure the content of soluble sugars; the Coomassie Brilliant Blue method to measure the content of soluble proteins; the spectrophotometric method to measure the content of chlorophyll; and the sulfosalicylic acid method to measure the content of free proline. Finally, we used the conductivity method to measure the conductivity of the cells.

Qualitative determination of hydrogen peroxide (H_2_O_2_): We prepared a 1 mg/mL solution of o-phenylenediamine (DAB) in 2 mL of ultrapure water (pH = 3.8). Then, we soaked 1 cm fresh rice leaf sections in the prepared solution for 10 min (under vacuum at 60 kPa pressure). We then incubated the leaf sections at room temperature for 10 min, placed the incubated leaf sections in anhydrous ethanol, and heated them in a water bath at 100 °C until the leaves turned white. The leaves were then observed under a microscope at 10× magnification.

Determination of plant elements: In the determination of plant elements, we used the microwave digestion–flame atomic absorption method to measure the content of metal elements such as Mg and Ga. At the same time, we used the barium sulfate turbidimetric method to measure the content of the S element.

Data analysis: The experimental data were analyzed using SPSS 17.0, and significant differences were analyzed using ANOVA. Comparisons between groups were indicated by uppercase letters, and comparisons within groups were indicated by lowercase letters. Different letters indicate statistically significant differences (*p* < 0.05), while the same letters or no letters indicate no statistically significant differences (*p* > 0.05).

## 5. Conclusions

Compared with the matrix materials, the crystal structures of rPLM, gPLM, and bPLM doped with Eu changed. The particle sizes of all three are in the micron range, with gPLM having a uniform morphology, while the other two are irregularly shaped structures. This complex structure and irregular crystal morphology do not have a significant impact on the germination potential and germination rate of rice seeds, but they do significantly inhibit the root and stem length of seedlings, destroy the cell structure of the roots, and reduce the biomass of seedlings. Due to the toxic sulfide substrate of rPLM, it can reduce the root vitality of seedlings and inhibit the accumulation of osmotic regulating substances and the synthesis of chlorophyll at concentrations of 10~100 mg/L. However, gPLM and bPLM can enhance the root vitality of seedlings and increase the content of osmotic regulation substances and chlorophyll to a certain extent. All three PLMs can cause an excessive amount of ROS in rice and damage the structure of the cell membrane. It is speculated that oxidative damage is the main toxic mechanism affecting rice growth.

Due to the unique afterglow characteristics of PLMs, in addition to paying attention to the plant toxicity caused by excessive accumulation of PLMs in water bodies or soil, the light stress on plants caused by the nocturnal afterglow of PLMs cannot be ignored. This will also be the focus of future research.

## Figures and Tables

**Table 1:** Summary of morphological characteristics of three kinds of PLMs.

Material	Morphology	Size	Zeta
rPLM	Irregular Block	30 ± 5 μm	−2.96 ± 2.29 mV
gPLM	Angular Trihedron	15 ± 3 μm	−13.18 ± 3.17 mV
bPLM	Irregular Block	40 ± 8 μm	−10.8 ± 0.92 mVa

**Table 2:** Effects of three kinds of PLMs with different concentrations on rice seed germination.

(mg/L)	rPLM		gPLM		bPLM	
	Germination Potential (%)	Germination Rate (%)	Germination Potential (%)	Germination Rate (%)	Germination Potential (%)	Germination Rate (%)
0	82.22 ± 0.08	93.33 ± 0.05	86.67 ± 0.01	93.33 ± 0.05	93.33 ± 0.05	91.11 ± 0.06
10	77.78 ± 0.03	95.56 ± 0.06	88.89 ± 0.03	91.11 ± 0.08	82.22 ± 0.03	93.33 ± 0.01
20	83.33 ± 0.39	95.56 ± 0.03	84.44 ± 0.03	93.33 ± 0.05	86.67 ± 0.05	93.33 ± 0.05
50	80.00 ± 0.05	93.33	88.89 ± 0.06	95.56 ± 0.06	82.22 ± 0.08	95.56 ± 0.003
100	90.00 ± 0.42	93.33 ± 0.03	82.22 ± 0.08	93.33 ± 0.05	91.11 ± 0.03	95.56 ± 0.03

## Data Availability

The data that support the findings of this study are available from the corresponding author upon reasonable request.
